# Derivation of chicken induced pluripotent stem cells tolerant to Newcastle disease virus-induced lysis through multiple rounds of infection

**DOI:** 10.1186/s12985-016-0659-3

**Published:** 2016-12-05

**Authors:** Leonardo Susta, Ying He, Jessica M. Hutcheson, Yangqing Lu, Franklin D. West, Steven L. Stice, Ping Yu, Zaid Abdo, Claudio L. Afonso

**Affiliations:** 1 US National Poultry Research Center, Exotic and Emerging Avian Viral Diseases Research Unit, Southeast Poultry Research Laboratory, Athens, GA 30605 USA; 2 Regenerative Bioscience Center, University of Georgia, Athens, GA 30602 USA; 3 Department of Animal and Dairy Science, University of Georgia, Athens, GA 30602 USA; 4 Department of Microbiology, Immunology, and Pathology, College of Veterinary Medicine and Biomedical Sciences, Colorado State University, Fort Collins, CO 80523 USA; 5 Present address: Department of Pathobiology, Ontario Veterinary College, University of Guelph, Guelph, ON N1G 2 W1 Canada; 6 Present address: College of Animal Science and Technology, Guangxi University, Nanning, Guangxi 53004 China

**Keywords:** Newcastle disease virus, Newcastle disease, ciPSCs, Infection, Tolerance, Cell count, Cell viability, RNA-seq, Pathways analysis

## Abstract

**Background:**

Newcastle disease (ND), caused by Newcastle disease virus (NDV), is a devastating disease of poultry and wild birds. ND is prevented by rigorous biocontainment and vaccination. One potential approach to prevent spread of the virus is production of birds that show innate resistance to NDV-caused disease. Induced pluripotent stem cell (iPSC) technology allows adult cells to be reprogrammed into an embryonic stem cell-like state capable of contributing to live offspring and passing on unique traits in a number of species. Recently, iPSC approaches have been successfully applied to avian cells. If chicken induced pluripotent stem cells (ciPSCs) are genetically or epigenetically modified to resist NDV infection, it may be possible to generate ND resistant poultry. There is limited information on the potential of ciPSCs to be infected by NDV, or the capacity of these cells to become resistant to infection. The aim of the present work was to assess the characteristics of the interaction between NDV and ciPSCs, and to develop a selection method that would increase tolerance of these cells to NDV-induced cellular damage.

**Results:**

Results showed that ciPSCs were permissive to infection with NDV, and susceptible to virus-mediated cell death. Since ciPSCs that survived infection demonstrated the ability to recover quickly, we devised a system to select surviving cells through multiple infection rounds with NDV. ciPSCs that sustained 9 consecutive infections had a statistically significant increase in survival (up to 36 times) compared to never-infected ciPSCs upon NDV infection (tolerant cells). Increased survival was not caused by a loss of permissiveness to NDV replication. RNA sequencing followed by enrichment pathway analysis showed that numerous metabolic pathways where differentially regulated between tolerant and never-infected ciPSCs.

**Conclusions:**

Results demonstrate that ciPSCs are permissive to NDV infection and become increasingly tolerant to NDV under selective pressure, indicating that this system could be applied to study mechanisms of cellular tolerance to NDV.

**Electronic supplementary material:**

The online version of this article (doi:10.1186/s12985-016-0659-3) contains supplementary material, which is available to authorized users.

## Background

Newcastle disease (ND) is a devastating disease of domestic poultry that causes severe economic losses both in developing and developed countries [1]. The disease is caused by virulent strains of Newcastle disease virus (NDV), an enveloped virus classified within the *Mononegavirales* order, *Paramyxoviridae* family, *Avulavirus* genus [2]. All NDV strains belong to a single serotype (avian paramyxovirus serotype 1, APMV-1), and the virus genome constitutes of a non-segmented, negative sense RNA molecule of approximately 15.2 Kb, which encodes for six structural proteins, namely from 3’ to 5’: nucleoprotein (NP), phosphoprotein (P), matrix (M), fusion (F), hemagglutinin-neuraminidase (HN), and polymerase (L) [3]. According to international standards, NDV strains can be classified as virulent or non-virulent, based on the intra cerebral pathogenicity index (ICPI), and on the deduced amino acid sequence of the F protein at amino acid residues 112 to 117 (cleavage site) [4].

Worldwide control of ND is carried out by costly and rigorous biocontainment and vaccination programs [1]. NDV vaccines protect birds against clinical signs, however do not confer sterile immunity, leading to circulation of virulent strains among vaccinated birds [5, 6]. Unrestrained virus circulation leads to virus evolution and ultimately emergence of new NDV strains [7]. Attempts to select for resistance against NDV in poultry through traditional breeding strategies have not been successful [8], and to date no poultry species susceptible to NDV have been successfully bred for increased resistance against development of ND. Production of poultry species that are resistant to NDV infection at the cellular level could hypothetically lead to the development of new means of controlling ND, especially in areas where ND is endemic and difficult to eradicate.

Induced pluripotent stem cell (iPSC) technology is a system by which adult cells such as skin fibroblast can be reprogrammed into an embryonic state, almost identical to embryonic stem cells. iPSCs can be utilized to generate animals with unique genetic and epigenetic traits as they can form germline competent chimeric animals and ultimately offspring with the specified phenotypes [9, 10]. iPSC technology has been successfully applied to mammalian species, including humans [9–12], and it has garnered success with avian species as well, such as chickens and quails [13–15]. Chicken induced pluripotent stem cells (ciPSCs) display characteristics indicative of a stem cell state including morphological and functional characteristic [13, 14, 16]. ciPSCs have demonstrable alkaline phosphatase enzymatic activity, and positive cytochemical staining for periodic acid-schiff (PAS) [13, 14, 16], consistent with stem cell staining characteristics [17]. As the most stringent proof of pluripotency, ciPSCs can be used to generate chimeric birds by transplantation into the embryo at early stages of embryogenesis, as shown with chicken-quails and chicken-chicken chimeras [13, 14]. Chimeric animals can then be bred to produce offspring with specific traits, as demonstrated by our group in other livestock species [15, 18]. Further, our laboratory has demonstrated that ciPSCs can undergo in vitro differentiation into early primordial germ cells (PGCs), which are capable of migrating to the gonads when injected into a developing embryo [19]. These ciPSC-PGCs can produce viable spermatozoa or oocytes, potentially leading to generation of fully transgenic offspring though crossbreeding of chimeric birds. With a similar mechanism, ciPSCs that are resistant to NDV could be used to generate transgenic birds that maintain the resistance trait in every tissue of the body.

Complete cellular resistance to NDV infection has been demonstrated in mammalian cells lacking specific sialylated moieties (mainly gangliosides, and N-syalilated glycoproteins) on the cell surface (absence of receptors) [20], while increased tolerance to NDV-induced cell death in cell culture has been associated with the presence of specific intracellular pathogen sensors – such as Retinoic Acid-Inducible Gene I (RIG-I) [21, 22]; increased type-I interferon response [23, 24]; and activity of the Mx protein family [25–27]. Also viral interference, although not a cellular resistance *per se*, inhibits NDV superinfection [28–30]. For the purposes of this study, the term *tolerance* was defined as the decreased susceptibility of ciPSCs to NDV-induced cell death, while still permissive to infection. In this context, “tolerance” differs from “resistance” to virus infection, which is considered as the total inability of the virus to infect cells (true resistance, receptor-mediated).

Infection of non-pluripotent cell lines with viruses at high multiplicity - without prior mutagenesis approach - has led to spontaneous selection of cells that are resistant or less susceptible to the cytolytic effect of the challenge virus. This has been reported for murine FM3A cells infected with NDV [31], porcine kidney cells line (ESK-R) infected with Influenza Virus [32]; hamster BHK-21 or CHO-K1 cells infected with murine encephalomyelitis virus or Ebola virus, respectively [33, 34]; canine (MDCK), swine (PK-15) and rabbit (RK-13) cell lines infected with cytolytic strains of Bovine Viral Diarrhea Virus (BVDV) [35]. A similar approach could theoretically produce spontaneously-induced resistance in ciPSCs, or generate ciPSCs less susceptible (tolerant) to NDV-induced cellular damage.

The aim of the present work was to evaluate the susceptibility of ciPSCs to infection with NDV of different virulence, and to establish a screening/selection method to naturally derive ciPSCs that are tolerant to NDV infection. Preliminary characterization of the interaction between NDV and ciPSCs, as well as investigation into the mechanisms of resistance, offers the opportunity to better characterize these cells and fits within the overarching effort to generate ND-resistant chickens.

## Results

### NDV replicates in ciPSCs

Chicken iPSCs were derived as previously described [16]. ciPSCs had typical characteristics of a stem cell fate: large nucleus to cytoplasm ratio, large nucleoli, positive cytochemical reaction for PAS and Alkaline Phosphatase, and expression of embryonic stem cell markers as shown by RT-PCR and immunocytochemistry [16]. Derived cells were named BA3NI, and expanded for the purpose of the experiments in this work.

**Fig. 1:**
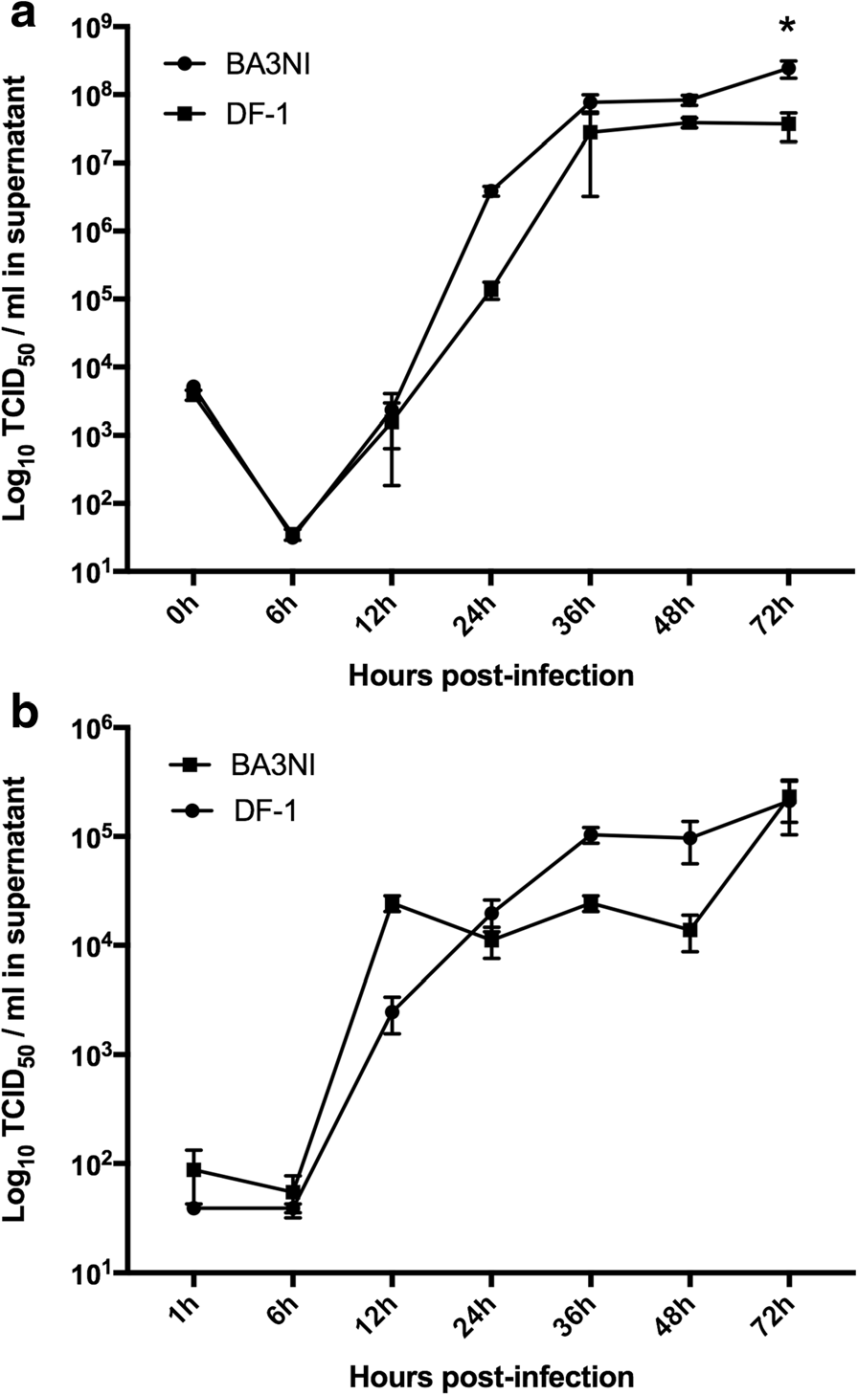
NDV Successfully replicates in ciPSCs. Multi-cycle replication kinetics of NDV in BA3NI ciPSCs, compared to DF-1 cells. Both NDV strains rZJ1-GFP (**a**) and rLS-RFP (**b**) are able to replicate in BA3NI cells and to be released in the supernatant at high titers. For each experiment, *n* = 3. Titers are represented as mean ± SD standard deviation (SD). *indicates significant differences between averaged titers at each time point (two-way ANOVA for repeated measures, Sidak’s test for multiple comparisons, *p* < 0.05)

To evaluate the ability of NDV to replicate in ciPSCs, multi-cycle virus growth curves were performed in BA3NI cells infected with both non-virulent (recombinant LaSota-RFP, (rLS-RFP) [29]) and virulent recombinant ZJ1-GFP (rZJ1-GFP) [36] NDV strains, and compared to growth in DF-1 cells (immortalized chicken fibroblast cells [37]). Briefly, both BA3NI and DF-1 cells were plated into 6-well-plates and infected with each virus at a multiplicity of infection (MOI) of 0.01. Virus growth at established time points [6, 12, 24, 36, 48, 72 h post-infection (hpi)] was measured by titration of the virus in the supernatant through limiting dilution using DF-1 cells, and expressed as Tissue Culture Infectious Dose 50% (TCID_50_)/ml. Titration results (Fig. 1, panel a and b) showed that the two viruses grew similarly in both ciPSCs and DF-1 cells up to 48 hpi, however, at 72 hpi rZJ1-GFP strain grew at significantly higher titers in ciPSCs compared to DF-1 cells (two-way ANOVA, Sidak’s test for multiple comparisons). As expected, the virulent strain showed a higher yield (2–3 logarithmic units) than the non-virulent strain in the same cell line. At 72 hpi, yields (mean ± SD, expressed as log_10_ TCID_50_ units/ml) in ciPSCs were 8.4 (+0.08, −0.10) and 5.36 (+0.22, −0.30) for rZJ1-GFP and rLS-RFP, respectively, while for DF-1 cells were 7.57 (+0.13, −0.17) and 5.32 (+0.25, −0.39) for rZJ1-GFP and rLS-RFP, respectively. Taken together, these data show that NDV is able to replicate in pluripotent BA3NI cells, slightly more efficiently than in DF-1 cells.

### NDV effectively induces cell death in ciPSCs

**Fig. 2:**
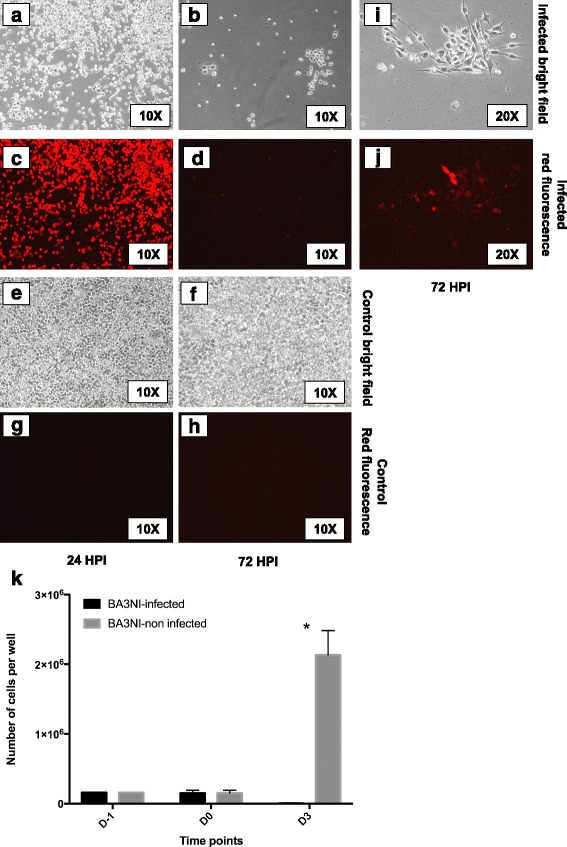
NDV effectively induces cell death in ciPSCs. Infected BA3NI cells show increasingly severe cytopathic effect from 24 hpi (panel **a**) to 72 hpi (panels **b**), which is associated with RFP fluorescence (panels **c** and **d**). Non-infected controls at the same time are confluent (panels **e** and **f**) and do not show RFP fluorescence (panels **g** and **h**). At 72 hpi, the few surviving cells left do not show cytopathic effect (Panel **i**). In infected cells, fluorescence has the highest intensity at 24 hpi, and subsides at 72 hpi in surviving cells (panel **d** and **j**). A representative counting of infected compared to non-infected BA3NI cells is shown in the graph (panel **k**). D-1, day of cell plating; D0, time of infection; D3, 72 hpi (day 3 post infection). For each experiment, *n* = 3. Counting values are expressed as mean ± SD. *indicates significant differences between average number of cells at each time point (two-way ANOVA for repeated measurements, Sidak’s test for multiple comparisons, *p* < 0.05)

The ability of the non-virulent rLS-RFP to induce cell death in BA3NI was assessed utilizing a high MOI. Briefly, BA3NI were plated at a density of 1.6×10^5^ cells/well in a 6-well-plate (time point: D-1) and the following day (24 h) infected with rLS-RFP at MOI = 50 (time point: D0). At 24 hpi, cells started showing severe cytopathic effect characterized by rounding and detachment from the plate (Fig. 2, panel a), which progressed at 72 hpi (Fig. 2, panel b). These changes were associated with intense fluorescence at 24 hpi (Fig. 2, panel c), which subsided at 72 hpi in viable cells (Fig. 2, panel d). Surviving cells at 72 hpi showed no signs of cytopathic effect, although some residual fluorescence could be observed (Fig. 2, panels i–j). A representative count of infected BA3NI cells compared to non-infected cells at 72 hpi (time point: D3) is presented (Fig. 2, panel k).

Based on five biological replicates, the percentage of viable BA3NI cells after infection, as counted at 72 hpi, was 15.52% ± 10.50 (mean ± SD) of the number of cells counted at the time of infection (D0), and 2.02% ± 2.25% (mean ± SD) of the control (non-infected) cells at D3 (72 hpi). Taken together, these data show that high MOI of rLS-RFP can be used to effectively induce cell death and select BA3NI cells.

### ciPSCs demonstrate rapid recovery after infection with NDV

**Fig. 3:**
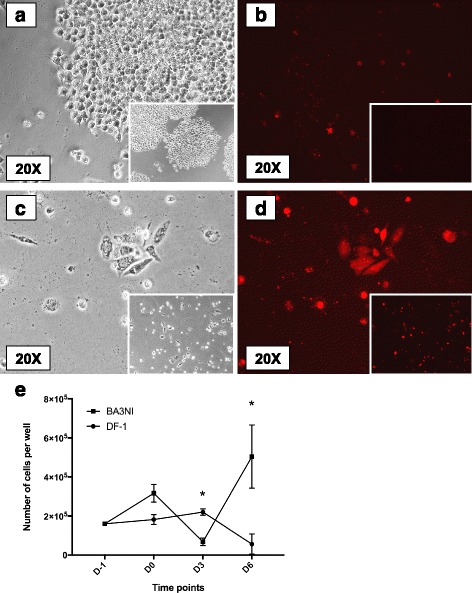
ciPSCs demonstrate rapid recovery after infection with NDV. Photomicrographs showing BA3NI (panels **a**, **b**) and DF-1 (panels **c**, **d**) cells at day 6 post-infection (144 hpi) with rLS-RFP at MOI = 50. BA3NI appear as large confluent colonies, as opposed to DF-1, which do not increase in number after infection. Panels **a**–**d**, magnification 20X; insets, magnification 10X; panels **a**, **c** bright field, **b**, **d** red fluorescence. A representative counting of infected DF-1 compared to infected BA3NI is shown in the graph (panel **e**). D-1, day of cell plating; D0, time of infection; D3, 72 hpi (day 3 post-infection); D6, 144 hpi (day 6 post-infection). For each experiment, *n* = 3. Counting values are expressed as mean ± SD. *indicates significant differences between average number of cells at each time point (two-way ANOVA for repeated measurements, Sidak’s test for multiple comparisons, *p* < 0.05)

The ability of BA3NI to recover after infection was assessed by counting the amount of viable cells at day 6 post-infection (144 hpi). BA3NI and DF-1 were plated into 6-well plates and infected at high MOI as described in the previous section. The number of viable cells present in the plate after infection was counted at 72 hpi and 144 hpi. Results showed that BA3NI and DF-1 cells recover at very different rates after infection. After markedly declining in number at 72 hpi, BA3NI cells were able to clear the virus and to proliferate as single large colonies that eventually became confluent by 144 hpi (Fig. 3, panel a). Conversely, DF-1 cells failed to recover and proliferate by 144 hpi (Fig. 3, panel c). Additionally, ciPSCs were able to clear the RFP fluorescence (Fig. 3, panel b), whereas DF-1 remained intensely fluorescent (Fig. 3, panel d). A representative count of the differences in recovery abilities between BA3NI and DF-1 is reported in (Fig. 3, panel e).

### Repeated NDV selection decreases susceptibility of ciPSC to NDV-induced cell death

Given the ability of ciPSCs to be infected by NDV and to quickly recover after infection, a screening system was devised to test the hypothesis that ciPSCs could become incrementally more *tolerant* (less susceptible) to NDV-induced cell death upon multiple rounds of infection/selection. Tolerance, for the purpose of our study, is a quantitative character assessed in a population of infected cells, and it is quantified by calculating the ratio between amount of viable cells present at 72 hpi (D3) and those present at the time of infection (0 h, D0); the higher the ratio, the more tolerant cells are deemed. Therefore, increased tolerance encompasses both the ability of cells to withstand virus-induced cell death, and to recover from it (clearing the infection and replicating).

**Fig. 4:**
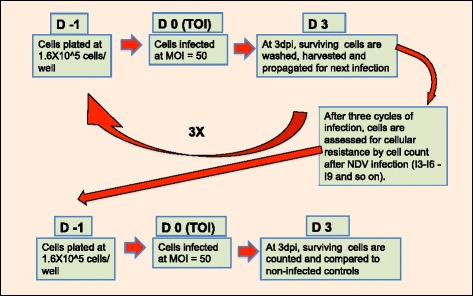
Iterative process for ciPSCs selection. At D-1 cells were plated at 1.6X10^5^ cells/well, and infected at D0 (time of infection, TOI) with NDV strain LS-RFP at MOI = 50. At D3 (3 days post infection [dpi]), cells were washed, expanded and propagated for another round of infection. Every 3 rounds of infection, a representative sample of the selected cells was infected with the same methodology to assess putative progression of tolerance

BA3NI cells were infected with rLS-RFP (MOI = 50), and surviving cells were propagated for the next infection; this was repeated nine times (see methods). After each round, surviving cells were named with increasing cardinal numbers (i.e., BA3NI-I1 indicates those cells that survived and were expanded after one round of infection: from round 0 to 1). After every 3 rounds of infection (rounds 3, 6, and 9), subsets of selected cells were used to assess putative progression of tolerance by counting surviving cells at 72 hpi. A schematic representation of the selection process is presented in Fig. 4.

**Fig. 5:**
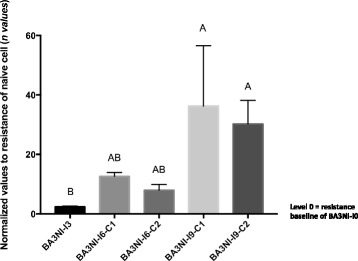
Repeated NDV selection decreases susceptibility of ciPSC to NDV-induced cell death. Progression of tolerance to rLS-RFP challenge (MOI = 50) between different rounds of infection (round 3, I3; round 6, I6; round 9, I9). C1 and C2 indicate the two clones sorted from the persistently infected cells (I6-PI). Survival data were obtained by normalizing to the basal level of tolerance of naïve cells (*n values*, see material and methods). BA3NI-I3, I6 and I9 showed increased tolerance with progressive rounds of infection, compared to never-infected BA3NI-I0 (level 0). For BA3NI-I9-C1 and C2, *n* = 9; for the other groups, *n* = 3. *n values* are expressed as mean ± SD. Letters indicate significant differences between groups (Kruskal-Wallis and Dunn’s method for multiple comparisons, *p* < 0.05)

**Fig. 6:**
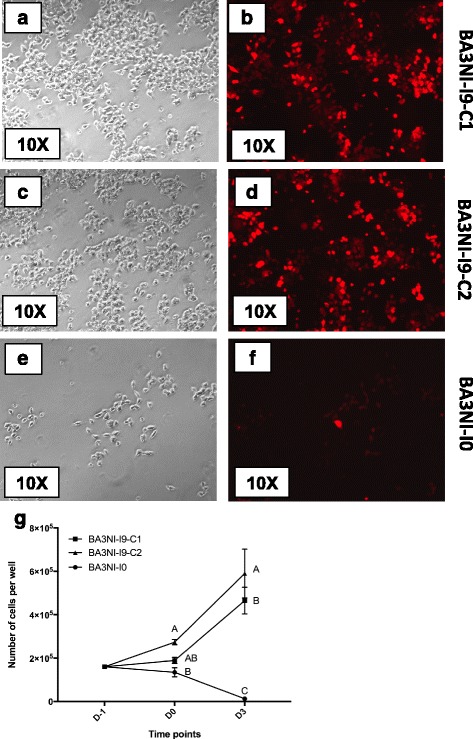
Multiple rounds of selection with NDV decrease susceptibility of BA3NI to NDV cytolytic effect at 72 hpi. After nine rounds of selection, BA3NI-C1-I9 (**a**, **b**) and BA3NI-C2-I9 (**c**, **d**) show decreased susceptibility to NDV cytolytic effect compared to BA3NI-I0 (never infected cells) (**e**, **f**). Cell counting shows that BA3NI-C1-I9 and BA3NI-C2-I9 have roughly 36 and 45 times more cells than naïve infected cells at 72hpi (**g**). D-1, day of cell plating; D0, time of infection; D3, 72hpi (day 3 post infection). For each experiment, *n* = 3. Counting values are expressed as mean ± SD. Letters indicate significantly different values at the same time point (two-way ANOVA for repeated measurements, Tukey’s test for multiple comparisons, *p* < 0.05)

Progression of tolerance between different rounds of infection was evaluated by normalizing the survival data for each round of infection to the basal level of tolerance of the naïve cells (*n values*, see material and methods). BA3NI-I3 (3rd round of infection), BA3NI-I6-clone (C)1 and C2 (the two clones sorted after the 6th round of infection, see next section) and BA3NI-I9-C1 and C2 (after 9th round of infection) showed increased resistance, when compared to never-infected BA3NI-I0 (*n values*: BA3NI-I3, 2.34 ± 0.26; BA3NI-I6-C1, 12.61 ± 1.31; BA3NI-I6-C2, 7.94 ± 1.95; BA3NI-I9-C1, 36.24 ± 20.30; BA3NI-I9-C2, 30.26 ± 7.90; mean ± SD) (Fig. 5). Using the non-parametric Kruskal-Wallis test followed by the Dunn’s test for multiple comparisons, differences in survival between BA3NI-I3 and B3NI-I9-C1 and C2 were considered to be statistically significant (*p* < 0.05). Representative pictures and cell counts of BA3NI-I9-C1 and C2 (selected for 9 times) upon infection are showed in Fig. 6. Upon infection with rLS-RFP at MOI = 50, at 72 hpi BA3NI-I9-C1 and C2 showed increased numbers of surviving cells compared to infected BA3NI-I0, as determined by microscopic evaluation (Fig. 6, panels a–f) and counting (Fig. 6, panel g). Noticeably, even as cells became progressively more tolerant to NDV infection, the virus was still able to infect the cells at each round, as shown by microscopic observation under fluorescence (Fig. 6, panels b, d).

### Nine-time selected ciPSCs are devoid of NDV

**Fig. 7:**
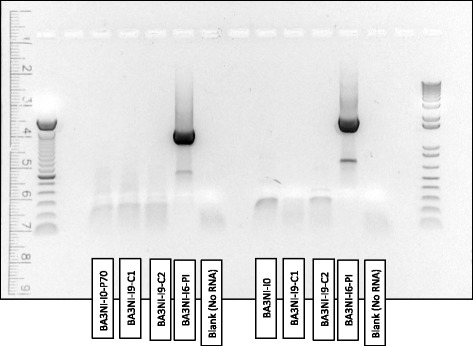
Nine-time selected BA3NI are devoid of NDV. Both BA3NI-I9-C1 and C2 (nine-time infected) are devoid of NDV genetic material, as assessed by RT-PCR for NDV Nucleoprotein (NP) and Polymerase (L) genes. See text for explanation

After the 6th round of infection, cells became persistently infected (BA3NI-I6-PI) and continuously expressed RFP fluorescence. To select for RFP negative cells, and to continue the selection process, non-fluorescent cells were sorted (Fluorescence Activated Cells [FACs] sorting) and 2 clonally-derived cell lines were propagated (BA3NI-I6-C1 and C2) to continue the selection to the 9th round of infection (see material and methods). At the end of the selection process, non-infected BA3NI-I9-C1 and C2 did not show fluorescence under microscopic observation. To confirm absence of residual NDV after the process of infection/selection, RT-PCR for NDV NP and L genes was performed on cellular RNA extracted from BA3NI-I9-C1 and C2 (the clones resulted at the end of the selection process, see material and methods). Expected product size was 1306 bp and 1761 bp for NP and L gene, respectively. Results showed absence of cDNA amplicons for RNA samples extracted from BA3NI-I9-C1, C2 and BA3NI-I0 cells (Fig. 7). Expected bands were observed when RNA from BA3NI-I6-PI cells was used as template. To confirm the negative RT-PCR results, a virus isolation (VI) test was performed on BA3NI-I9-C1 and C2 pellets (one passage in egg) [38, 39]. Negative results for VI (data not shown) were in agreement with the RT-PCR results, and confirmed that cells at the end of the selection process were devoid of residual virus.

### Increased tolerance to virus-induced cell death in selected ciPSCs is not caused by decreased permissiveness to NDV infection

**Fig. 8:**
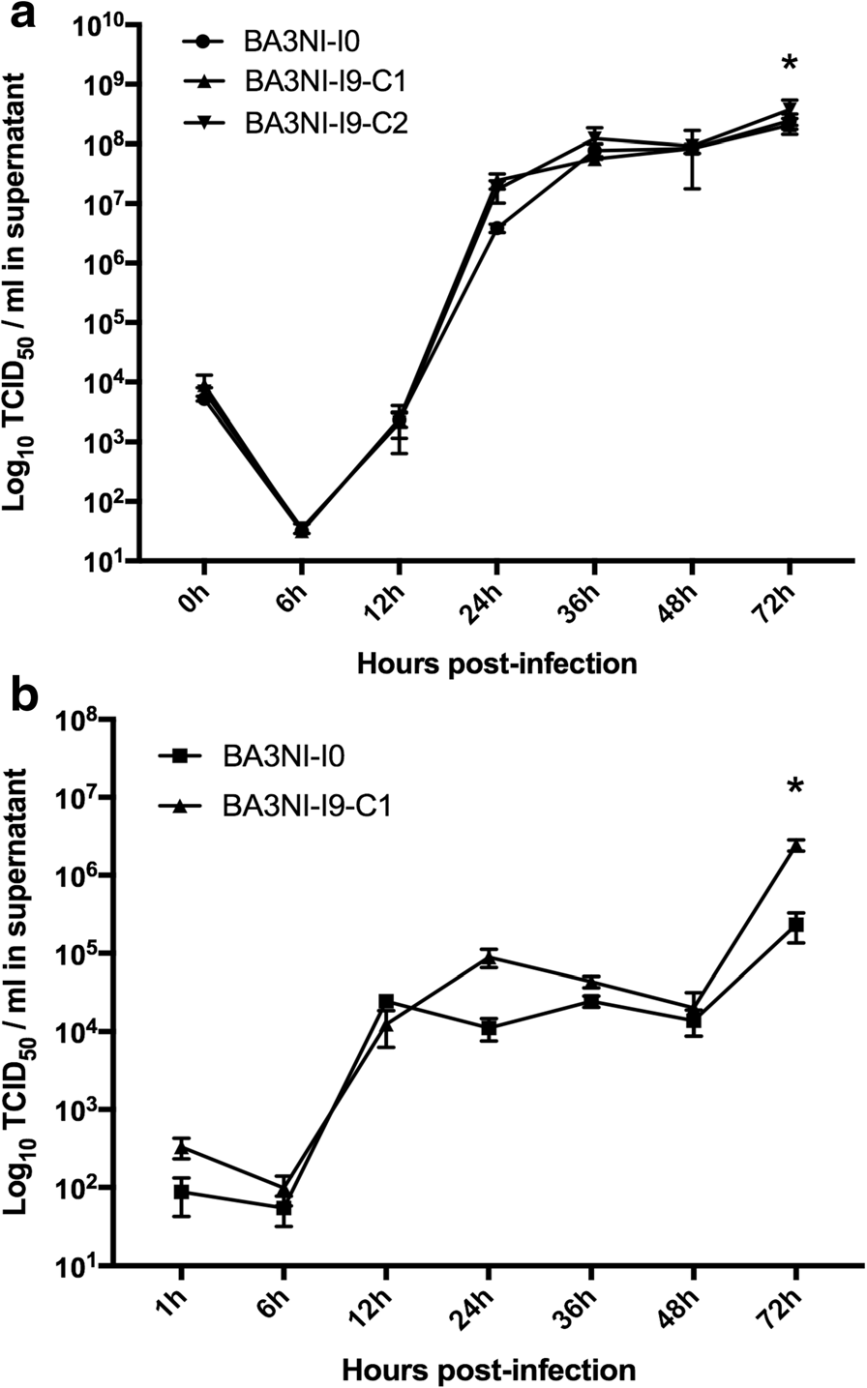
Nine-time selected ciPSCs are permissive to NDV replication. To determine if BA3NI at the end of the selection process were less permissive than naïve (never selected) BA3NI cells, multi-cycle growth curves were performed in BA3NI-C1 and C2 (selected) and BA3NI-I0 (naïve) with both virulent and non-virulent NDV strain rZJ1-GFP (**a**) and rLS-RFP (**b**). *n* = 3, titers expressed as mean ± SD. *indicates significant differences between average titers at each time point (two-way ANOVA for repeated measures, Tukey’s test for multiple comparisons, *p* < 0.05)

To evaluate if the increased tolerance to virus-induced cell death observed with BA3NI-I9 C1 and C2 was caused by a decreased permissiveness to NDV infection, multi-cycle growth curves were performed on BA3NI-I0 and BA3NI-I9-C1 and C2 with non-virulent and virulent NDV strains rLS-RFP and rZJ1-GFP, as described previously. At 72hpi, strains rLS-RFP and rZJ1-GFP yielded higher titers in BA3NI-I9-C1 and C2, respectively, compared to BA3NI-I0 cells. At other time points (6, 12, 24, 36, 48hpi) no differences in virus yield between cell lines were observed (Fig. 8, panel a and b). These data indicate that increased tolerance of BA3NI-I9-C1 and C2 cells to NDV infection was not caused by a decreased permissiveness to infection, since virus yield in BA3NI-C1 or C2 is slightly higher than in naïve cells (BA3NI-I0).

### Differential gene expression between BA3NI-I0 and BA3NI-I9

RNA sequencing (RNA-seq) was used to investigate the effect of NDV selection on the BA3NI transcriptome, comparing the naïve BA3NI-I0, BA3NI-I9-C1 and BA3NI-I6-PI (persistently infected cells). Extracted RNA (in biological triplicates) for each cell line was reverse-transcribed with random priming, and sequenced using an Illumina 1.9 platform. Table 1 shows the starting and ending number of reads with the minimum average quality per sample, and the average coverage provided by each sample measured against the reference genome along with the range of read-lengths after the filtering process. Paired ends were matched, and only lengths greater than or equal to 75 nucleotides were used in the following analyses. Differential RNA expression was analyzed using the Tuxedo Suite [40–42] software, with a 0.05 significance cutoff. Results were summarized using cummeRbund software package, and false discovery rate, as implemented in Cuffdiff [41, 43], was used to correct for multiple comparisons. The analysis identified 62, 869 and 1009 differentially expressed genes between BA3NI-I0 and I6, BA3NI-I6 and I9, and BA3NI-I0 and I9, respectively (see Additional file 1: Tables S1, Additional file 2: Table S2 and Additional file 3: Table S3 for details, gene names and annotations). Pathway analysis was carried out with PathView analysis using the KEGG pathways. Significantly upregulated and downregulated pathways between comparisons are reported in Table 2.

**Table 1:** Summary statistics of the sequencing reads performed in the Illumina platform

Sample	Initial read count	Final read count (%)	Minimum mean QC (Phred)	Range of fragment length
BA3NI-I0	1,155,428	817,773 (70.8%)	27	75–136
BA3NI-I6-PI	1,052,378	793,149 (75.4%)	27	75–136
BA3NI-I9	1,083,813	799,653 (73.8%)	27	75–136

Reads were preprocessed by trimming 15 nucleotides from the 5’ end, and then by trimming both ends using a sliding window of 3 nucleotides and an average Phred score of 25. This was followed by filtering reads such that 80% or more nucleotides had quality score over 20 and removing all reads with length less than 75

**Table 2:** List of pathways upregulated and downregulated between selected, persistently infected, and naïve BA3NI ciPSCs

BA3NI-I9-C1 compared to BA3NI-I0		BA3NI-I6 compared to BA3NI-I9-C1		BA3NI-I6 compared to BA3NI-I0	
Upregulated	Downregulated	Upregulated	Downregulated	Upregulated	Downregulated
Al, ASP, GLU Metabolism	Fatty Acid Elongation	Fatty Acid Elongation	Al, ASP, GLU Metabolism		Ribosome Biogenesis in Eukaryotes
Val, Leu, Iso degradation	Steroid Hormone Biosynthesis	Steroid Hormone Biosynthesis	Lysine degradation		Spliceosome
Lysine degradation	Oxidative Phosphorylation	Oxidative Phosphorylation	Inositol Phosphate Metabolism		
Inositol Phosphate Metabolism	Pyrimidine Metabolism	Pyrimidine Metabolism	mRNA surveillance Pathway		
MAPK Signalling Pathway	Glutathione Metabolism	Arginin and Proline Metabolism	MAPK Signalling Pathway		
ERBB Signalling Pathway	N-Glycan Biosynthesis	Tyrosine Metabolism	ERBB Signalling Pathway		
FOXO Signalling Pathway	GPI-anchor Biosynthesis	Glutathione Metabolism	FOXO Signalling Pathway		
PI Signalling System	Ribosome Biogenesis in Eukaryotes	N-Glycan Biosynthesis	PI Signalling System		
Oocyte Meiosis	Ribosome	Glycosaminoglycan Degradation	Oocyte Meiosis		
Ubiquitin-Mediated Proteolysis	RNA polymerase	GPI Anchor Biosynthesis	Ubiquitin-Mediated Proteolysis		
Endocytosis	Spliceosome	Arachidonic Acid and Metabolism	Endocytosis		
mTOR signalling Pathway	Proteasome	Metabolism of Xenobiotics by Cytochrome P450	mTOR Signalling Pathway		
Apoptosis	Protein Export	Ribosome	Apoptosis		
Adrenergic signalling in Cardiomyocytes	Neuroactive ligand-receptor interaction	Proteasome	Adrenergic signalling in Cardiomyocytes		
Vascular smooth muscle contraction	Protein Processing in Endoplasmic Reticulum	Protein Export	Vascular smooth muscle contraction		
WNT Signalling Pathway	Lysosome	Neuroactive Ligand-Receptor Interaction	WNT Signalling Pathway		
Dorso-Ventral Axis Formation	Phagosome	Protein Processing in Endoplasmic reticulum	Dorso-Ventral Axis Formation		
VEGF Signalling Pathway	Cardiac Muscle Contraction	Lysosome	VEGF Signalling Pathway		
Focal Adhesion	Cell Adhesion Molecules	Cardiac Muscle Contraction	Focal Adhesion		
Tight Junction		Cell Adhesion Molecules	Adherens Junction		
Gap Junction			Tight Junction		
Regulation of Actin Cytoskeleton			Gap Junction		
Insulin Signalling Pathway			Toll-like receptor Signalling pathway		
GnRH Signalling Pathway			Jak-Stat Signalling pathway		
Progesterone-mediated Oocyte maturation			Regulation of Actin Cytoskeleton		
			Insulin Signalling pathway		
			GnRH Signalling Pathway		
			Progesterone-Mediated Oocyte Maturation		
			Salmonella Infection		
			Influenza A infection		

## Discussion

In the present work we demonstrated the susceptibility of ciPSCs to infection with low and high virulence NDV strains, and established a selection protocol to generate ciPSCs increasingly tolerant to NDV-induced cell death. Our data demonstrated that ciPSCs can support productive infection with virulent and non-virulent NDV strains, and viral release in the supernatant to levels higher than those reached in an immortalized fibroblast cell line (DF-1). This is in agreement to what recently reported with a different ciPSC line created utilizing the same technology [44], suggesting that susceptibility to NDV infection in ciPSCs is likely a well-conserved feature. However, permissiveness to virus replication is a characteristic that avian stem cells may not share with other species. In fact, human and murine pluripotent stem cells were non-permissive to infection with multiple viruses, such as hepatitis C virus (HCV) [45], varicella zoster virus (VZV) [46], and only moderately permissive to Influenza A virus and human Herpes-Simplex virus-1 [47]. At least for HCV and VZV, permissiveness to infection appeared to increase with the degree of cellular differentiation [45, 46]. This might suggest that, contrary to what observed with other viruses, NDV infection is not particularly restricted by the degree of cellular differentiation, or that chicken iPSCs have specific characteristics that are suitable for virus growth. The mechanism underlying this fast recovery of ciPSCs, in contrast to DF-1, after infection with high MOI remains to be elucidated.

Selection of ciPSCs after 9 rounds of infection allowed for the derivation of a ciPSCs line that is up to 36 times more tolerant to NDV infection than naïve ciPSCs. Despite increased tolerance, selected cells remained permissive to NDV infection. Both rLS-RFP and rZJ1-GFP grew at higher titers in 9-time selected compared to never-selected ciPSCs, possibly reflecting the higher number of cells available for virus replication at later time points. Since selected cells were still permissive to NDV replication and susceptible to the virus-induced cytopathic effect, we opted to use the operational definition of *tolerance*, rather than the term *resistance*.

The mechanisms of increased tolerance in selected cells are unclear and await further characterization. Progression of tolerance in this study was evaluated by assessing the number of cells present in the plate at 72 hpi compared to the number of cells present at the time of infection at 0 hrs. Therefore, increased tolerance can be a combination of decreased susceptibility to cell-death, or heightened recovery rate after infection, including virus clearance and replicative efficacy. Doubling time in nine-time selected ciPSCs lines, however, did not appear to be shorter compared to naïve ciPSCs (data not shown), suggesting that a faster replication is not the main mechanism leading to a more tolerant state.

RNA sequencing showed that several genes are differentially expressed between selected and non-selected cells. Pathway analysis revealed that most of the genes upregulated in BA3NI-I9-C1 included those involved in basic cellular processes, such as amino acid metabolism, cellular proliferation, apoptosis, cell adhesion and signalling, and regulation of the cytoskeleton. In agreement with our results, a recent study showed that basic cellular processes were also differentially expressed in the lungs of partially resistant Fayoumi chickens infected with AIV, when compared to fully susceptible Leghorn chickens [48]. In particular, genes involved in *actin filament-based movement* and *multicellular organismal process* were upregulated, while those involved in *cell adhesions (CAM)* and *immune response* were downregulated [48].

During the selection process, ciPSCs became persistently infected after the 6th round of infection. It is possible that the high MOI used during the selection process might have led to production of defective interfering particles facilitating the establishment of a persistent infection [28]. However, sorting of non-fluorescent cells was sufficient to rescue clones that were not persistently infected, demonstrating that increased tolerance observed with BA3NI-I9-C1 and C2 cells was not the consequence of viral interference.

As they have the potential to produce chimeric and fully transgenic birds [19], ciPCSc that are either resistant or less susceptible to NDV infection could be used to produce transgenic birds that are resistant to ND at a cellular level. The present study is a preliminary investigation toward this direction. Our results show that increased tolerance of ciPSCs is a complex mechanism, and it is difficult to predict if this trait could be maintained through development without being silenced and be successfully passed onto offspring. Future studies should aim at inducing higher levels of resistance in selected cells, possibly using transgenic approaches where a single locus (e.g., small interfering RNA against virus genes) could confer resistance with a simpler mechanism. In this study a transgenic approach was avoided, as spontaneously resistant cells were sought through multiple selection events.

## Conclusions

In this study, we have demonstrated that ciPSCs are susceptible to infection with high- and low-virulence NDV, and have developed a system for derivation of cell lines that are incrementally more tolerant to NDV infection through multiple rounds of infection and selection. The system described in this study could be utilized to study host mechanism of tolerance to infection against NDV in vitro, and to develop new platforms for production of disease-resistant livestock through the use iPSCs in birds and other species.

## Methods

### Cells

Detailed methodology for derivation of ciPSCs from CEFs are reported elsewhere [16]. Briefly, primary chicken embryo fibroblasts from black Australorp chickens embryos were reprogrammed with Minicircle vectors expressing human transcription factors POUF51, NANOG51, SOX2, and LIN28. BA3NI cells were maintained in cKSR (knock-out serum replacement) media [DMEM/F12 (Gibco), supplemented with 20% knockout serum replacement (KSR; Gibco), 2 mM L-glutamine (Gibco), 0.1 mM nonessential amino acids (Gibco), 1xPen/strep(Gibco), 0.1 mM β-mercaptoethanol (Sigma- Aldrich), and 10 ng/mL basic fibroblast growth factor (bFGF; R&D Systems)] in pre-coated plates to increase cell adhesion [Matrigel (BD Biosciences); diluted 1:100 in DMEM/F12]. For passaging, cells were dissociated with Accutase (Innovative Life Technologies). DF-1 cells (chicken fibroblast cells line - ATCC CRL 12203) were maintained in fibroblast media [Dulbecco’s modified Eagle’s medium, high glucose (Hyclone) with 10% fetal bovine serum (Hyclone), 4 mM L-glutamine (Gibco) and 1x Pen/strep (Gibco)], and dissociated upon confluence with 0.05% trypsin. All cells were maintained at 37C and 5% CO_2_.

### Eggs and chickens

Embryonated chicken eggs utilized for production of virus stock and virus isolation were obtained from the Southeast Poultry Research Laboratory (SEPRL) Specific Pathogen Free (SPF) white Leghorn flock.

### Viruses

Recombinant NDV strains ZJ1-GFP (rZJ1-GFP) and LaSota-RFP (rLS-RFP) were obtained from the SEPRL virus repository. rZJ1-GFP is a virulent strain with ICPI of 1.88 carrying the Green Fluorescent Protein (GFP) gene between the P and M genes [36]. rLS-RFP is a lentogenic NDV with ICPI of 0.0 carrying a Red Fluorescent Protein gene between the F and the HN genes [29]. Each virus expresses the respective fluorescent genes upon infection in cell cultures. Insertion of the extra gene cassette in the virus genomes did not induce significant decrease of virus fitness compared to parental strains, as assessed by multi-cycle growth curves in cell culture [29, 36]. To produce virus stock, viruses were inoculated in the chorioallantoic cavity of 9 to 10 day-old embryonating SPF eggs. Dead eggs, or eggs surviving after 5 days of incubation were chilled at 4 C for 24 h, and hemagglutination (HA) - positive allantoic fluid was extracted, pooled, clarified by centrifugation (10’ at 5000 rpm), and stored in 1 ml aliquots at -80C [38, 39]. Virus stock was titered in DF-1 cells in 96-well plates through limiting dilutions, and titer was expressed as Tissue Culture Infection Dose 50% (TCID_50_)/ml using the Spearman-Karber method [49].

### Growth Curves in ciPSCs

To assess the ability of NDV to replicate in ciPSCs, a multi-cycle growth assay (multiplicity of infection, MOI = 0.01) was performed in BA3NI-I0 and compared to NDV replication in DF-1 cells. Additionally, growth curves were performed to compare virus growth in BA3NI-I0 and BA3NI-I9-C1 and C2 after selection. Cells (BA3NI-I, BA3NI-I9-C1 and C2 and DF-1) were plated in triplicate into 6-well-plates at a density of 7×10^5^ cells/well. After 24 h, media was discarded and cells were infected with 0.01 MOI of either rLS-RFP or rZJ1-GFP diluted in 1%-serum fibroblast media or 5% KSR media (1 ml inoculum/well), respectively for infection of DF-1 or ciPS cells. After 1 h absorption, cells were washed three times with PBS and then incubated with maintenance fibroblast or KSR media. At time points 0, 6, 12, 24, 36, 48, and 72 hpi, 200 μL of supernatant were collected and replaced with fresh media. The amount of virus in the supernatant samples was titrated in DF-1 cells using 96-well-plates through limiting dilutions, and expressed as TCID_50_/ml using the Spearman-Karber method [49]. For infection with rLS-RFP, maintenance media during infection was supplemented with 8.5 μg/ml porcine trypsin (Sigma), to allow cleavage of the fusion protein and multiple cycles of infection [50]. Concentration of trypsin was optimized in preliminary experiments to increase virus production and minimize any possible cell damage; a concentration of 8.5 μg/ml did not cause detachment or negatively affect viability of ciPSCs or DF-1 cells (data not shown).

### Assessment of cytolytic effect of NDV on ciPSCs

Preliminary experiments were carried out to optimize the best conditions to assure highest levels of NDV-induced ciPSCs death. Marked cell death was observed at 72 hpi using a high MOI (=50) in combination with low cell density (initial plating density =1.6×10^5^ cells/35-mm dish). These conditions were maintained for all the infection procedures of this work. Briefly, cells were infected in triplicate with rLS-RFP at MOI = 50, in 6-well-plate format. Cells were plated at 1.6×10^5^ cells/well, and after 24 h cells were infected with rLS-RFP diluted in 5% KSR, as described above. After 1 h absorption, the infectious inoculum was discarded and replaced with cKSR. Three wells were used as mock-infected control and treated with 1 ml of 5% KSR without virus. Photomicrographs (Nikon Eclipse TE300, SPOT Software 5.0) were taken every 24, 48, and 72 hpi. To assess the amount of remaining cells after infection, counting was performed at 72 hpi, as follows. Wells were washed with PBS and adherent cells were dissociated with 1 ml Accutase (Innovative Cells Technologies) for 4 min at 37C. After neutralization of accutase with 1 ml of KSR media, cells were transferred in conical tubes and centrifuged at 1400 rpm for 5 min. Cell pellet was resuspended with 300 μL or 500 μL maintenance media (depending on the amount of cells), and the number of viable cells was determined with an automated cell counter (Nexcelom Bioscience Cellometer Auto T4), using 1:1 staining with 0.02% Trypan Blue solution (Sigma) for viability assessment.

### Assessment of recovery after NDV infection

The ability of BA3NI, compared to DF-1, to recover after infection was assessed by counting cells at 72 and 144 hpi. BA3NI-I0 and DF-1 were plated in triplicate into 6-well plates at 1.6×10^5^ cells/well, and after 24 h infected with rLS-RFP at MOI = 50, as described above. At the time of infection, 3 extra well for each cell line were counted and averaged to effectively calculate the exact MOI. Mock infected DF-1 and BA3NI cells were incubated with reduced serum media, as described above. At 72 and 144 hpi, 3 wells for each cell lines (both mock and infected) were counted using the Nexcelom automatic counter, as described above.

### Development of a screening system to select ciPSCs after multiple rounds of infection with rLS-RFP NDV

Based on the ability of NDV to cause cytolytic effect in ciPSCs, a screening system was developed to assess the ability of ciPSCs to become incrementally tolerant to NDV upon multiple rounds of infection/selection (Fig. 4). The day before infection (D-1), BA3NI cells that were never infected previously (BA3NI-Infection (I) 0) were plated at 1.6X10^5^ cells/well in a 6-well-plate format. The following day (time of infection, TOI, D0) cells were infected with rLS-RFP at MOI = 50, as described above. At 72 hpi (end of selection, D3), cells were washed with PBS, expanded and passaged until no observable red fluorescence was detectable under microscopic observation. Cells rescued after each round of infection were named with increasing cardinal numbers (i.e., BA3NI-I1 indicates those cells that survived and were expanded after one round of infection). Cells expanded from the previous round of infection were then plated and infected as described. Every 3 rounds of infections (rounds 3, 6, and 9) a batch of selected cells was used to assess putative progression of tolerance by counting surviving cells at 72 hpi with the Nexcelom automated counter, as described in the previous section.

Tolerance was calculated by normalizing the number of remaining infected cells at 72 hpi (D3), over the number of cells at the time of infection (D0), which was directly counted to assess the correct MOI at the time of infection. To evaluate the progression of tolerance between different rounds of infection, and to decrease inter-experimental variability, the normalized values of tolerance of BA3NI-I3, I6 and I9 derived from each experiment were divided to the normalized baseline tolerance values of the BA3NI-I0 controls that was run in parallel each time. These double-normalized values (*n values*) were compared between each other (Fig. 5).

### Persistent infection and FAC sorting

After the 6th round of infection, BA3NI-I6 became persistently infected (PI). In order to eliminate the virus from the cell population and to proceed in the screening process, the non-fluorescent cells were separated by FAC sorting. Briefly, BA3NI-I6 cells were dissociated with Accutase, centrifuged for 5’ at 1400 rpm, and washed with PBS twice in order to obtain a final concentration of about 1×10^6^ cells/ml. Resuspended cells in PBS were sorted using a Beckman Coulter MoFlo XDP machine, gated for non-fluorescent cells. Of the several colonies that were negative for RFP, two were expanded (BA3NI-I6-C1 and C2), and underwent the selection process through rounds 6 to 9.

### RNA extraction and RT-PCR

To determine the presence of NDV in cells that underwent the whole selection process (BA3NI-I9-C1 and C2), RT-PCR for two NDV genes (NP, genomic position 43–1349; and L, genomic positions 13338–15099) was performed on total RNA. Cellular RNA was extracted as standard protocols using a phenol-chloroform system. RT-PCR was carried out using the SuperScript III One-Step RT-PCR System with Platinum Taq DNA polymerase kit (Invitrogen, Carlsbad, CA). Primer sequences are available upon request.

### Virus isolation from ciPSCs

To determine the presence of NDV in ciPSCs that were persistently infected, and underwent 9 rounds of infection/selection, VI was performed on BA3NI-I9-C1 and BA3NI-I9-C2, using BA3NI-I0 as negative control. Cells from one confluent 100 mm dish for each cell line were harvested by dissociation with Accutase and centrifuged at 1400 rpm for 5 min after neutralization with cKSR media. Supernatant was discarded; the resulting cell pellet resuspended in 1 ml cKSR media, and the number of cells/ml was counted (Nexcelom automated counter). To free cell-associated virus, cells were freeze-thawed three times at -80C, and treated with oscillating sonicator for 2 min on ice. The homogenized pellet was used to perform virus isolation and titration in eggs, using standard methods [38, 39].

### Deep Sequencing data and differential gene expression between BA3NI-I0, BA3NI-I6, and BA3NI-I9

#### RNA-Seq data assembly and properties

RNA was extracted in biological triplicate from 3×10^6^ BA3NI-I0, BA3NI-I6-PI, and BA3NI-I9-C1 cells using the with RNeasy Mini kit (Qiagen), following the manufacturer protocol. RNA sample quality was assessed through a Bioanalyzer (Agilent). RNA libraries were created from each RNA sample, as follows. Starting with total RNA, first strand cDNA amplification was performed using Oilgo(dT) oligomers. A cDNA library was then created using random hexamer primers, and millions of short DNA reads were generated by sequencing the RNA samples in individual lanes of an Illumina 1.9 instrument (Illumina) by the University of Georgia Genomic Facility. Data were preprocessed by first trimming the first 15 nucleotides from each of the 5’ end of each of the reads and then using a sliding window of size 3 and an average quality of 25 to trim both ends of the reads. This was followed by a filtering step to remove reads such that 80% or more nucleotides within a read had a quality score greater than 20. FastQC was used to examine the quality of the reads before and after trimming and indicates that on average all of the retained reads were of average quality greater than 19 for all samples. Paired end were matched and only lengths greater than or equal to 75 nucleotides were used in the following analyses.

#### RNA-Seq analysis of differential expression

The Tuxedo Suite [41–43] was used to evaluate the transcriptome of BA3NI-I0, BA3NI-I6-PI, and BA3NI-I9 with one sample obtained for each condition. All reads were aligned to the Chicken (*Gallus Gallus*) genome obtained from http://useast.ensembl.org/info/data/ftp/index.html (last accessed July 29th 2016). The protocol for differential gene expression analysis was published elsewhere [43]. Briefly, we first built the indexed genome using bowtie 2. Then, utilizing this genome and the annotated genes, we first aligned the Illumina sequences using TopHat 2 and assessed differential expression using Cufflinks 2 (significance, *p* < 0.05). We used the commeRbund package in R for visualization and further analysis.

### Statistical analysis

Differences between mean *n values* were analyzed using the Kruskal-Wallis test followed by the Dunn’s test for multiple comparisons. Virus titers and cell counts over time were analyzed using a two-way analysis of variance (ANOVA) for repeated measures followed by a Tukey’s or Sidak’s test for multiple comparisons. All tests were considered statistically significant for *p* values < 0.05. Statistical analysis was carried out using GraphPad Prism for iOS, version 7 (GraphPad Software, La Jolla California USA).

## Additional files

Additional file 1: Table S1. List of differentially expressed genes between BA3NI-I0 and BA3NI-I6 (average of biological triplicates). (XLSX 57 kb)
Additional file 2: Table S2. List of differentially expressed genes between BA3NI-I6 and BA3NI-I9-C1 (average of biological triplicates). (XLSX 189 kb)
Additional file 3: Table S3. List of differentially expressed genes between BA3NI-I0 and BA3NI-I9-C1 (average of biological triplicates). (XLSX 214 kb)

## Abbreviations

ANOVA: Analysis of variance
ciPSCs: Chicken induced pluripotent stem cells
dpi: Days post infection
hpi: Hours post infection
iPSCs: Induced pluripotent stem cells
MOI: Multiplicity of infection
ND: Newcastle disease
NDV: Newcastle disease virus
